# Trastuzumab Specific Epitope Evaluation as a Predictive and Prognostic Biomarker in Gastric Cancer Patients

**DOI:** 10.3390/biom9120782

**Published:** 2019-11-26

**Authors:** Jiwon Koh, Soo Kyung Nam, Youn Woo Lee, Jin Won Kim, Keun-Wook Lee, Chan-Young Ock, Do-Youn Oh, Sang-Hoon Ahn, Hyung-Ho Kim, Keon-Wook Kang, Woo Ho Kim, Ho-Young Lee, Hye Seung Lee

**Affiliations:** 1 Department of Pathology, Seoul National University Hospital, Seoul National University College of Medicine, Seoul 110-744, Korea; jiwonsophia@gmail.com (J.K.); woohokim@snu.ac.kr (W.H.K.); 2 Department of Pathology, Seoul National University Bundang Hospital, Seoul National University College of Medicine, Seongnam-si 13488, Korea; chris3017@naver.com; 3 Department of Nuclear Medicine, Seoul National University Bundang Hospital, Seoul National University College of Medicine, Seongnam-si 13488, Korea; younwoolee66@gmail.com; 4 Department of Internal Medicine, Seoul National University Bundang Hospital, Seoul National University College of Medicine, Seongnam-si 13488, Korea; whunt@daum.net (J.W.K.); hmodoctor@snubh.org (K.-W.L.); 5 Department of Internal Medicine, Seoul National University Hospital, Seoul National University College of Medicine, Seoul 110-744, Korea; ock.chanyoung@gmail.com (C.-Y.O.); ohdoyoun@snu.ac.kr (D.-Y.O.); 6 Department of Surgery, Seoul National University Bundang Hospital, Seoul National University College of Medicine, Seongnam-si 13488, Korea; viscaria@snubh.org (S.-H.A.); hhkim@snubh.org (H.-H.K.); 7 Department of Nuclear Medicine, Seoul National University Hospital, Seoul National University College of Medicine, Seoul 110-744, Korea; kangkw@snu.ac.kr

**Keywords:** trastuzumab, HER2, gastric cancer, prognosis, prediction

## Abstract

While human epidermal growth factor receptor 2 (HER2) immunohistochemistry (IHC) antibodies bind to the intracellular domain, trastuzumab binds to the extracellular epitope of HER2 receptor: target of drug action. We aimed to evaluate clinical significance of the new IHC method assessing the target of trastuzumab in gastric cancer (GC) patients and compare with conventional methods. Sixty-nine trastuzumab-treated GC patients were enrolled from two different cohorts. Additionally, we enrolled 528 consecutive GC patients to evaluate prognostic implications of HER2 test methods. HER2 status was assessed by trastuzumab IHC, HER2 IHC (4B5), and HER2 silver in situ hybridization (SISH). HER2 IHC showed 3+ in 48/69 trastuzumab-treated patients (69.6%), however, trastuzumab IHC showed 3+ in 25 (36.2%). Patients with trastuzumab IHC ≥2+ had significantly better progression-free survival (PFS) and overall survival (OS) than their counterpart (*p* = 0.014). In univariate analysis, trastuzumab IHC ≥2+ and HER2 IHC 3+ were only significant predictive factors for OS in trastuzumab-treated patients. Of the 528 consecutive GCs, patients with trastuzumab IHC ≥2+ had shorter disease-free survival (DFS) and OS (*p* = 0.008 and 0.031, respectively), while conventional methods failed to reveal any significant survival differences. HER2 assessment by trastuzumab IHC was different from conventional HER2 test results. Trastuzumab IHC was suggested to be a significant predictive factor for trastuzumab responsiveness and prognostic factor for consecutive GCs.

## 1. Introduction

Gastric cancer (GC) is one of the leading causes of cancer-related deaths worldwide [1]. Especially for advanced stage cases, previously reported survival rates have been less than 45%, despite implementation of multiple chemotherapeutic agents [2,3]. There have been numerous researches to identify therapeutic targets for GC, including recent discoveries related to human epidermal growth factor receptor 2 (HER2). *HER2* amplification and HER2 protein overexpression are observed in 6%–35% of GCs [4,5]. Although *HER2* over-expressing status is consistently reported as a poor prognostic factor in breast cancer [6,7], the prognostic role of HER2 in GC remains controversial [8,9,10].

Trastuzumab is the first humanized anti-HER2 monoclonal antibody and is widely used as a targeted therapy for HER2-positive breast cancer [6]. Following the success of trastuzumab for a GC (ToGA) trial in 2010 [11], trastuzumab-based therapy has become the standard therapy for HER2-overexpressing gastric cancer [12]. Therefore, evaluating *HER2* status became important for treatment decisions to achieve better clinical outcomes [13]. In light of its clinical implications, various methods of assessing *HER2* status have been developed, including HER2 immunohistochemistry (IHC), fluorescence in situ hybridization (FISH), and silver in situ hybridization (SISH). Although no single method is a complete gold standard, HER2 IHC is the most widely used assessment technique [14] because of its convenience and availability.

Nonetheless, not all patients with pathologically confirmed HER2-positive status have survival benefit from trastuzumab therapy [15,16], and the overall response rate (ORR) ranges from 32% to 68% [13]. Many studies were performed in order to explain the unresponsiveness and resistance to trastuzumab. On a molecular basis, one of the suggested mechanisms of resistance is the activation of the PI3K pathway by de novo alteration or through direction interaction with HER3 protein [17]. Additionally, IGF1R overexpression or loss of *PTEN* tumor suppressor gene has been linked to the decreased sensitivity to trastuzumab [18,19]. Others have focused on intra-tumoral heterogeneity in HER2 overexpression and *HER2* gene activation; a study has shown that this heterogeneity can be observed in up to 74.0% of surgically resected cases of GC [20].

Compared with the mechanisms noted above, relatively less attention has given to the diagnostic modalities for *HER2* status. HER2 IHC is the most widely used method of choice, however, most of the commonly used commercially available antibodies for HER2 IHC bind intracellular region of HER2 protein near the C-terminal, while trastuzumab is designated to bind the extracellular epitope [21]. A constitutively active and truncated form of HER2 (p95-HER2) can be detected via HER2 IHC, but p95-HER2 does not harbor the binding site of trastuzumab [22]. Furthermore, cell surface proteins such as mucins restrict the access of trastuzumab to its epitope on the HER2 receptor, blocking the inhibitory actions of the drugs [23]. Therefore, HER2 IHC with commercially available antibodies may not accurately represent the interaction between trastuzumab and the HER2 receptor. A new IHC protocol utilizing trastuzumab itself, and therefore targeting the extracellular epitope, is needed to provide more precise predictions of the chemotherapeutic response to trastuzumab.

The aims of this study are to test (1) whether the results of trastuzumab IHC differ from the results of conventional HER2 IHC, (2) whether trastuzumab IHC has better performance for predicting the treatment outcome of trastuzumab-based therapy, and (3) the prognostic implication of trastuzumab IHC results in comparison with other *HER2* assessment methods such as HER2 IHC and *HER2* SISH.

## 2. Materials and Methods

### 2.1. Patients and Samples

A total of 69 patients diagnosed with GC and treated with a trastuzumab-based palliative treatment were studied; 37 patients were from Seoul National University Bundang Hospital (Seongnam-si, Republic of Korea; cohort 1) and 32 patients were treated in Seoul National University Hospital (Seoul, Republic of Korea; cohort 2). IHC for both HER2 and trastuzumab was performed using whole sections of formalin-fixed paraffin-embedded (FFPE) biopsy specimens or surgically resected specimens.

To test the prognostic significance of trastuzumab IHC in GC patients who were not treated with trastuzumab, we additionally studied a separate group of 528 consecutive stage I–IV GC patients treated at Seoul National University Bundang Hospital. All 528 patients had undergone surgical resection from 2003 to 2005, followed by conventional adjuvant or palliative treatment if clinically indicated. Representative 2-mm cores from surgical specimens were selected by an experienced gastrointestinal pathologist to construct tissue microarrays.

Clinicopathological data, including progression-free survival (PFS), disease-free survival (DFS), and overall survival (OS), were collected based on a retrospective review of medical records and pathologic reports. This study was approved by the institutional review boards of Seoul National University Hospital (1603-068-748) and Seoul National University Bundang Hospital (B-1603-338-305).

### 2.2. HER2 IHC and SISH

Automatic HER2 immunohistochemical staining was performed on 3-µm slides using PATHWAY anti-HER2/neu (4B5; rabbit monoclonal; pre-dilution; Ventana Medical Systems, Tucson, AZ, USA) antibody and an ultraView Universal DAB kit (Ventana Medical Systems, Tucson, AZ, USA) on an automatic immunostainer (BenchMark XT, Ventana Medical Systems, Tuscon, AZ, USA), following the manufacture’s guidelines. Automatic dual-color SISH of HER2 was performed using an automatic SISH staining device (BenchMark XT, Ventana Medical Systems, Tuscon, AZ, USA), according to the manufacturer’s protocols for INFORM HER2 DNA and INFORM Chromosome 17 (CEP17) probes (Ventana Medical Systems, Tuscon, AZ, USA). Signals from 20 tumor cells were counted and a HER2/CEP17 ratio ≥2.0 was classified as *HER2* gene amplification group.

### 2.3. Trastuzumab IHC

The manual staining protocol for trastuzumab IHC was as follows: 4-µm sections of FFPE GC tissue were deparaffinized and rehydrated, followed by antigen retrieval using proteinase K (Invitrogen, Carlsbad, CA, USA) at 37 °C for 10 min. After 10 min of hydrogen peroxide blocking, additional super blocking process and human-to-human blocking process were performed (Scytek Laboratories, Logan, UT, USA) for 5 min and 60 min, respectively, to enable visualization of the human monoclonal antibody—in this case the trastuzumab—on human tissue. Trastuzumab (10 µg/mL; Roche Pharma (Schweiz) Ltd., Reinach, Switzerland) was added as the primary antibody and incubated for 90 min, followed by goat anti-human IgG-horseradish peroxidase (HRP) as the secondary antibody for 20 min. 3,3’-Diaminobenzidine (DAKO, Carpinteria, CO, USA) was added, and nuclear counter-staining was performed using Mayer’s hematoxylin.

### 2.4. Evaluation of HER2 and Trastuzumab IHC

For both HER2 and trastuzumab IHC, we evaluated IHC staining intensity according to the recommendations from the DAKO HercepTestTM guidelines (DAKO) for GC [20,24] as follows: 0, no reactivity or membrane staining in <10% of tumor cells; 1+, faint/barely perceptible partial membrane staining in ≥10% of tumor cells; 2+, weak-to-moderate complete or basolateral membrane staining in ≥10% of tumor cells; and 3+, moderate-to-strong complete or basolateral membrane staining in ≥10% of tumor cells.

### 2.5. Validation Using GC Cell Line

We used SNU-1 *erbB2*+ gastric cancer cell line to validate that trastuzumab and HER2 IHC antibody bind to different epitopes. Trastuzumab and HER2 IHC antibody were labeled with quantum dot (Qdot^®^ 565 Antibody Labeling Kit). The quantum dot labeled antibody was loaded to incubated cell lines for one hour. After the washing out process, fluorescent images were acquired (LSM 800, Carl Zeiss). In addition, after loading 0.01 mg/mL of trastuzumab, quantum dot labeled antibodies were loaded and images were acquired.

### 2.6. Statistical Analysis

We calculated Spearman’s rank correlation coefficient to compare HER2 IHC with the new trastuzumab IHC. To assess the predictive value of the new IHC method in trastuzumab-treated patients, Kaplan–Meier survival analyses were performed for PFS and OS. The statistical significance of the survival differences was analyzed using the log-rank test. Univariate and multivariate Cox regression analyses were performed for both PFS and OS, and *p*-values <0.05 were considered to be statistically significant. Within the consecutive GC cohort, the results of HER2 IHC, trastuzumab IHC, and *HER2* SISH were compared using the Spearman rank test. For each method, prognostic associations with DFS and OS were studied using Kaplan–Meier survival analysis, as well as univariate and multivariate Cox regression analyses. All statistical analyses were performed with the SPSS Statistics 19.0 software package (SPSS Inc., Chicago, IL, USA).

## 3. Results

### 3.1. Clinicopathologjcal Characteristics of The Study Population

Clinicopathological characteristics of the study population with trastuzumab-based therapy are summarized in Table 1. The median age was 63 years, which included 51 (73.9%) men and 18 (26.1%) women. Of the 69 cases, 18 (26.1%) were biopsy specimens and 51 (73.9%) were surgically resected specimens. Most of the patients (91.3%) had advanced GC, and 35 (50.7%) patients had distant metastasis at baseline, and all of the patients were treated with trastuzumab-based therapy. The median PFS and OS durations were 9.8 (range 0.1–147.4) months and 21.6 (range 0.1–147.4) months, respectively. The patients from two separate institutes (cohort 1 and 2) did not differ significantly in terms of median age, sex, or baseline clinical status (data not shown).

### 3.2. Comparison between HER2 IHC and Trastuzumab IHC

**Figure 1:**
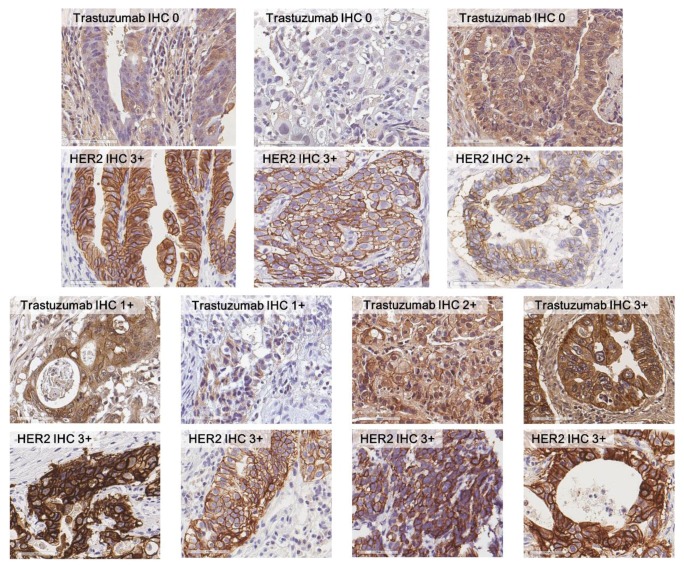
Comparison between HER2 IHC and trastuzumab IHC. Within HER2 IHC 2+ or 3+ group, the results of trastuzumab IHC varied.

Results of HER2 IHC and trastuzumab IHC are shown in Table 2 and Figure 1. The proportion of trastuzumab IHC 3+ was lower than that of HER2 3+ in the trastuzumab-treated group (36.2% versus 69.6%). Of the 48 HER2 3+ cases, only 24 (50.0%) cases were also 3+ in trastuzumab IHC; the proportion of cases that showed no membranous staining by trastuzumab IHC was as high as 31.3%. Fifteen (78.9%) out of 19 HER2 IHC 2+ cases were stained negative by trastuzumab IHC. The correlation between the two IHC methods was analyzed; the correlation coefficient between HER2 IHC and trastuzumab IHC was 0.466 (Spearman’s rho; *p*-value < 0.001).

### 3.3. Survival Analysis According to Trastuzumab IHC

**Figure 2:**
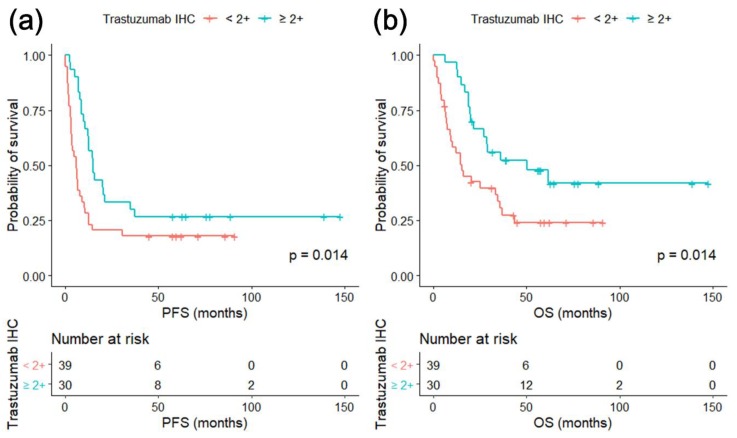
(**a**) Progression free survival (PFS) and (**b**) overall survival (OS) according to trastuzumab IHC results.

By applying trastuzumab IHC intensity scores, we subclassified the study population into two groups: trastuzumab IHC ≥2+ group and trastuzumab IHC <2+ group. Though all the patients were HER2 IHC 3+ or *HER2* FISH/SISH positive and trastuzumab-treated, the Kaplan–Meier survival analysis showed that significant survival differences existed within this cohort. Specifically, the trastuzumab IHC ≥2+ group had significantly better PFS (*p* = 0.014) and better OS (*p* = 0.014) than did the trastuzumab IHC <2+ group (Figure 2). Univariate analyses of OS showed that trastuzumab IHC ≥2+ and HER2 IHC 3+ were only significant predictive factors for OS in patients receiving trastuzumab therapy (Table 3).

Kaplan–Meier survival curves for PFS and OS are shown. Patients were categorized into either trastuzumab IHC <2+ group or ≥2+ group; PFS and OS were compared accordingly. Trastuzumab ≥2+ group had significantly better PFS and OS in trastuzumab-treated study population

### 3.4. HER2 Assessments in 528 Consecutive GC Patients

**Figure 3:**
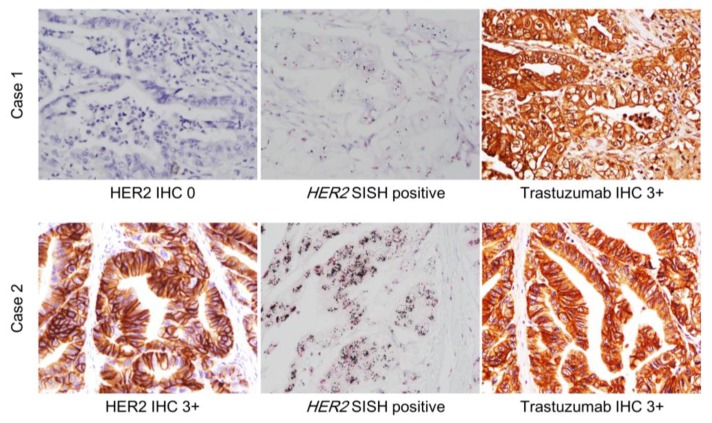
Results of HER2 IHC, HER2 silver in situ hybridization (SISH), and trastuzumab IHC in two cases among consecutive gastric cancer patients. Case 1 shows negativity by HER2 IHC, however, HER2 amplification was confirmed by SISH, and was 3+ by trastuzumab IHC. Case 2 showed 3+ by both HER2 and trastuzumab IHC, and HER2 gene amplification was observed as well.

Table 4 shows the clinicopathological characteristics of the consecutive gastric cancer patients. Among the 528 patients with various histologic types and stages of disease, 510 (95.1%) patients had negative HER2 status, determined based on HER2 IHC and HER2 SISH results. When HER2 IHC and trastuzumab IHC results were compared, of the 402 (75.0%) cases that showed negativity based on HER2 IHC, two cases were 3+ when using trastuzumab; one of these two cases showed HER2 amplification by HER2 SISH (Table S1 and Figure 3). Among patients with HER2 IHC 3+, we saw a similar pattern of discrepancy between HER2 IHC and trastuzumab IHC as had been observed in the previously mentioned cohorts of patients who received trastuzumab-based therapy: 16 (69.6%) out of 23 HER2 IHC 3+ cases showed negative staining by trastuzumab IHC, while five cases (21.7%) remained also 3+ by trastuzumab IHC. Variable trastuzumab IHC staining results were observed within the HER2 SISH amplification group; 79.5% of the cases that showed HER2 amplification by SISH stained negative when using trastuzumab IHC (Table S1).

**Figure 4:**
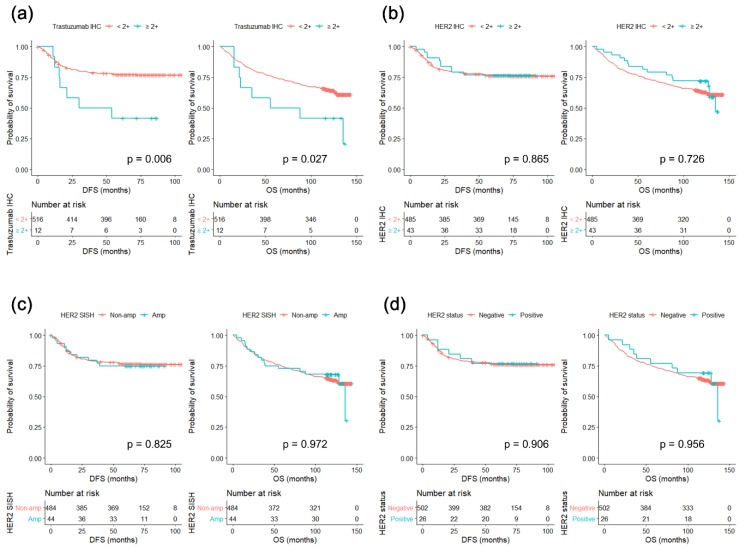
Disease-free survival (DFS) and overall survival (OS) of consecutive gastric cancer patients according to various HER2 assessment methods. Kaplan–Meier survival curves for both DFS and OS according to various subgroups are shown. (**a**) Trastuzumab IHC ≥2+ was associated with better survival with statistical significance. (**b**–**d**) Other HER2 assessment methods were not able to discriminate significant survival differences between subgroups.

Kaplan–Meier survival analyses using the log-rank method were performed for the two different IHC methods, *HER2* SISH results, and overall *HER2* status, as depicted in Figure 4. The trastuzumab IHC ≥2+ group had significantly better DFS and OS compared to trastuzumab IHC <2+ group (*p* = 0.006 and 0.027, respectively; Figure 4a). However, no significant survival differences were observed in the analyses of subgroups based on HER2 IHC, HER2 SISH, or overall HER2 status (using both IHC and SISH) (Figure 4b–d). In the Cox univariate analyses of DFS (Table 5), trastuzumab IHC ≥2+ was a significant poor prognostic factor (*p* = 0.008), as was the presence of lymphatic invasion, perineural invasion, vascular invasion, and advanced pTNM stage (stage III or IV). However, in the multivariate Cox regression analysis of DFS, trastuzumab IHC ≥2+ failed to be an independent prognostic factor. In univariate analyses of OS, trastuzumab IHC ≥2+ was also associated with poor survival (*p* = 0.031). None of the other methods of *HER2* status assessment had statistically significant prognostic associations in either the univariate or multivariate analyses of OS (Table 5). In concordance with the previously reported data, advanced age, the presence of vascular invasion, and pTNM stages III–IV turned out to be the independent prognostic factors for OS in the cohort of consecutive gastric cancer cases. Considering the very low incidence of HER2 positivity in consecutive gastric cancers, we combined the trastuzumab-treated cohort with the consecutive GC cohort to perform a multivariate survival analysis in the entire cohort. In this entire cohort, trastuzumab IHC was a significant poor prognostic factor for OS (*p* = 0.035) independent of age, sex, Lauren classification, pTNM stage, and HER2 IHC status (data not shown).

### 3.5. Validation of Different Epitopes for Each Antibody Using GC Cell Line

As shown in Figure S1, both trastuzumab and HER2 IHC antibody can detect SNU-1 cells by the quantum dot (QD) method. After incubating the cells with 0.01 mg/mL of trastuzumab, we found that trastuzumab-tailed QD could not detect the cells, while HER2 IHC antibody-tailed QD had no problem regarding tracing the cells. Taken together, we validated that cellular detection using trastuzumab itself may better represent the actual drug binding site, and thus may more accurately predict the actual mechanism of action.

## 4. Discussion

This is the first study utilizing trastuzumab as the primary antibody for immunohistochemical staining in GC. Based on the results of this study, we found that trastuzumab IHC may be a valuable tool for predicting the treatment outcome of trastuzumab-based therapy, and it is additionally a potential prognostic factor for general GC.

Targeted therapy using monoclonal antibodies has become the standard of care for certain types of cancer. Following the success of trastuzumab in *HER2*-amplified breast cancer, the concept of companion diagnostics emerged [25]. Early development of targeted agents relied on the assumption that clinical benefits from these agents would correlate with the clinically measurable expression of the target protein in tumor cells. The level of target expression is most commonly measured via immunohistochemical staining using commercially available antibodies that differ from the therapeutic monoclonal antibodies. However, not all companion diagnostic tests are clinically useful; clinical benefit of cetuximab treatment in metastatic colorectal carcinoma was not related to EGFR immunostaining results [26], and trastuzumab response rate in *HER2*-positive GC remains less than 50% [11]. One of the rational approaches to improve the companion diagnostics would be using the therapeutic antibodies themselves as immunostaining reagents; however, due to technical problems and cost-effectiveness issues, this approach is not widely implemented. In this study, we overcome the problematic nonspecific staining of background tissue related to the direct use of humanized antibody in IHC. Furthermore, we have successfully validated the use of trastuzumab itself as the primary antibody of immunostaining, and we have shown that this new method may potentially be a novel predictive marker for therapeutic response and a prognostic factor in GC.

**Figure 5:**
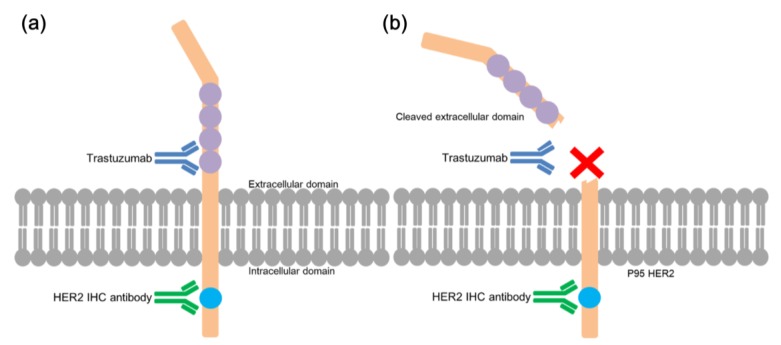
(**a**) Schematic illustration of different targets of HER2 IHC and trastuzumab IHC within HER2 protein. Widely-used antibodies for HER2 IHC bind to the intracellular region of HER2 protein near the C-terminal, while trastuzumab is designed to bind to the extracellular epitope. (**b**) When HER2 protein undergoes in vivo proteolytic cleavage, the extracellular domain is lost, therefore, trastuzumab cannot bind to HER2; however, theoretically antibodies for HER2 IHC can bind to the intracellular domain, producing positive IHC results.

By comparing new trastuzumab IHC with conventional HER2 IHC in the patients with HER2 IHC 3+ or *HER2* FISH/SISH positive and trastuzumab-treated, we observed significant discrepancies, most notably within the HER2 IHC 3+ group. Up to 39.6% of the HER2 IHC 3+ cases stained 0 or 1+ when using trastuzumab IHC. We concluded that this positivity rate of trastuzumab IHC among HER2 IHC 3+ patients is consistent with previously reported response rates to trastuzumab treatment [11]. One of the reasons for this discrepancy is the topological difference between the binding sites of trastuzumab and the antibody for HER2 IHC. HER2 is a type I transmembrane protein (185 kDa; p185HER2) composed of three domains: the extracellular domain, which harbors the trastuzumab binding site; the transmembrane domain; and the intracellular domain, which has functional tyrosine kinase domain and the C-terminal end of the protein. One of the unique features of HER2 protein is that it undergoes in vivo proteolytic cleavage of its extracellular domain (ECD), generating a truncated 95-kDa intracellular protein (also called p95-HER2) [22] (Figure 5). Most of the commonly used commercial antibodies for HER2 IHC (including the product used in this study) all bind to the intracellular domain near C-terminal end of HER2 [27]. Therefore, HER2 IHC represents both full-length HER2 and p95-HER2, even though p95-HER2-positive cells are expected to evade the anti-tumor effect of trastuzumab. In addition, another possible explanation for the discrepancy may be the hindering effect of cell surface mucins [23]. The mucin on tumor cell surface could inhibit the interaction between trastuzumab and its target epitope on the extracellular domain of the HER2 protein, while it would not affect the interaction of the HER2 immunostaining antibody and the intracellular domain of HER2.

In addition to the distinctive results of trastuzumab IHC, we were also able to observe significant differences in the treatment responses to trastuzumab-based therapy, according to trastuzumab IHC results. The 69 patients who were tested for trastuzumab IHC were the selected population for trastuzumab therapy using the best currently available modalities of *HER2* testing and were expected to benefit from the treatment. However, the significant difference in PFS between trastuzumab IHC ≥2+ group versus trastuzumab IHC <2+ group was observed, implying that trastuzumab IHC can more precisely identify the patients who would receive real benefits from trastuzumab therapy.

Although *HER2* status has constantly been a poor prognostic factor in numerous studies of breast cancer, the prognostic role of *HER2* status in GC is still unclear. Some studies have suggested that *HER2* status has no prognostic influence in GC [8,10,28,29], while other studies have suggested that positive *HER2* status is a poor prognostic factor [30,31,32,33]. We thought that one reason for these previous conflicting results was different definitions and assessment methods of *HER2* status: some studies have tested HER2 protein expression using IHC, while others relied on *HER2* gene expression status. Using tissue microarrays from 528 consecutive GC patients, we intended to compare the novel trastuzumab IHC with the previously used *HER2* assessment methods to determine whether trastuzumab IHC intensity scores were associated with the overall prognosis of gastric cancer. In the analyses of HER2 IHC, *HER2* SISH, *HER2* status (as determined by IHC and SISH), and trastuzumab IHC, statistically significant differences in DFS and OS were only evident for trastuzumab IHC, though trastuzumab IHC status was not an independent prognostic factor in the multivariate Cox regression analysis. Based on our current understanding, we have not been able to provide a clear explanation of the underlying biology why the patients’ prognosis only showed significant associations with trastuzumab IHC. However, we have concluded that trastuzumab IHC ≥2+ stands out as a potential poor prognostic factor in general GC patients without trastuzumab treatment. This conclusion is worth reporting because even the most widely used assessment methods of *HER2* assessment listed above all failed to discriminate any survival differences from the same study population.

The implementation of the new IHC method involved some technical issues. Most importantly, since trastuzumab is a humanized monoclonal antibody, anti-human IgG antibody must be incubated as the secondary antibody to visualize the binding of trastuzumab with the HER2 protein, which results in undesirable background staining. Despite this technical problem, some researchers attempted to implement direct trastuzumab IHC in breast cancer tissue. The first approach was direct biotin labeling of trastuzumab itself to avoid using the secondary antibody at all [34]. Although this approach was successful in reducing the background staining, some studies have suggested that biotinylation of primary antibodies may result in diminished affinity and specificity [35,36]. The second approach was implemented in breast cancer tissue by adding superblock and human-to-human block on the tissue before applying the primary antibody. This method succeeded in minimizing nonspecific background staining and maximizing the membranous staining amplification by the secondary antibody [37]. We implemented the superblock and human-to-human blocking process in our trastuzumab IHC protocol in a similar manner as described in the previous literature, which provided us with desirable IHC results. In addition to trastuzumab itself, there is a commercially available anti-HER2 antibody (clone SP3; Cell Marque, Rockllin, CA, USA) targeting extracellular epitope of HER2. If the background staining of trastuzumab IHC is the main source of pitfall hindering proper diagnosis, the assessment of performance of SP3 on predicting trastuzumab treatment outcome should be warranted and compared with trastuzumab IHC to finalize which one is the better method.

This study has the limitation of being a retrospective study that relied on a relatively small study population. However, considering the low percentage of *HER2* positivity in GC, the results of this study is valuable and worth reporting. In the future, a prospective study of trastuzumab IHC in an international multi-center setting would be necessary for further validation of this new method.

## 5. Conclusions

In the current era of targeted therapy, it is of enormous importance to identify and validate predictive markers as companion diagnostics for certain types of treatments. Furthermore, from the perspective of pathologists, measures to improve the quality of diagnosis are major concerns that deserve substantial attention. Trastuzumab IHC was designed in an effort to visualize the actual drug-binding site, thus enabling a better representation of the biological function of HER2 protein. Applying a standardized procedure on FFPE tissue, we were able to perform immunohistochemical staining of trastuzumab itself as the primary antibody. We suggest that trastuzumab IHC may be an excellent predictive factor for the treatment outcomes of trastuzumab-based therapy.

## Figures and Tables

**Table 1:** Clinicopathological characteristics of the study population with trastuzumab-based therapy.

	*n* (%)
Age	
Median (range)	63 (30–84)
Sex	
Male	51 (73.9%)
Female	18 (26.1%)
Specimen	
Biopsy	18 (26.1%)
Surgical resection	51 (73.9%)
Histology	
Intestinal	49 (71.0%)
Diffuse	15 (21.7%)
Mixed	5 (7.2%)
Clinical stage (baseline)	
Early gastric cancer	6 (8.7%)
Advanced gastric cancer	63 (91.3%)
Metastasis (baseline)	
Absent	34 (49.3%)
Present	35 (50.7%)
HER2 IHC status	
0	0 (0%)
1+	2 (2.9%) ^a^
2+	19 (27.5%)
3+	48 (69.6%)
Trastuzumab IHC status	
0	32 (46.4%)
1+	7 (10.1%)
2+	5 (7.2%)
3+	25 (36.2%)
PFS (months)	
Median (range)	9.8 (0.1–147.4)
OS (months)	
Median (range)	21.6 (0.1–147.4)
Total	69 (100%)

^a^ These two cases had HER2 amplification confirmed by separate fluorescent in situ hybridization test. Abbreviation: IHC, immunohistochemistry; PFS, progression-free survival; OS, overall survival.

**Table 2:** Comparison between human epidermal growth factor receptor 2 (HER2) immunohistochemistry (IHC) and trastuzumab IHC in trastuzumab-treated gastric cancer patients.

		Trastuzumab IHC				
		0	1+	2+	3+	Total
**HER2** **IHC**	0	0 (0%)	0 (0%)	0 (0%)	0 (0%)	0 (0%)
	1+	2 (100%)	0 (0%)	0 (0%)	0 (0%)	2 (2.9%)
	2+	15 (78.9%)	3 (15.8%)	0 (0%)	1 (5.3%)	19 (27.5%)
	3+	15 (31.3%)	4 (8.3%)	5 (10.4%)	24 (50.0%)	48 (69.6%)
	Total	32 (46.4%)	7 (10.1%)	5 (7.3%)	25 (36.2%)	69 (100.0%)

Abbreviation: IHC, immunohistochemistry.

**Table 3:** Univariate cox regression analysis of progression-free survival and overall survival in trastuzumab-treated population.

	Progression-Free Survival		Overall Survival	
	HR (95% CI)	*p*	HR (95% CI)	*p*
Age				
<65	1.00		1.00	
≥65	1.33 (0.78–2.28)	0.291	1.62 (0.89–2.95)	0.112
Sex				
Male	1.00		1.00	
Female	1.12 (0.61–2.07)	0.711	0.98 (0.50–1.95)	0.964
Histology				
Intestinal	1.00		1.00	
Diffuse	1.02 (0.52–2.00)		1.18 (0.57–2.41)	
Mixed	1.79 (0.70–4.57)	0.475	1.72 (0.60–4.90)	0.576
Clinical stage				
EGC	1.00		1.00	
AGC	1.62 (0.51–5.22)	0.413	2.40 (0.58–9.94)	0.226
HER2 IHC				
<2+	1.00		1.00	
≥2+	0.53 (0.13–2.21)	0.383	0.79 (0.19–3.29)	0.749
HER2 IHC				
1+, 2+	1.00		1.00	
3+	0.23 (0.13–0.44)	< 0.001	0.29 (0.15–0.55)	<0.001
Trastuzumab IHC				
<2+	1.00		1.00	
≥2+	0.51 (0.30–0.88)	0.016	0.47 (0.25–0.87)	0.016
Metastasis				
No	1.00		1.00	
Yes	1.79 (1.03–3.11)	0.038	1.52 (0.83–2.79)	0.180

Abbreviations: HR, hazard ratio; CI, confidence interval; *p*, *p*-value; EGC, early gastric cancer; AGC, advanced gastric cancer; IHC, immunohistochemistry.

**Table 4:** Clinicopathologic characteristics of consecutive gastric cancer patients according to HER2 status.

	*HER2* Negative	*HER2* Positive	*p*-Value
Age			
<65	306 (61.0%)	13 (50.0%)	0.265
≥65	196 (39.0%)	13 (50.0%)	
Sex			
Male	334 (66.5%)	18 (69.2%)	0.776
Female	168 (33.5%)	8 (30.8%)	
Location			
Lower	256 (51.0%)	15 (57.7%)	0.444
Middle	153 (30.5%)	7 (26.9%)	
Upper	78 (15.5%)	4 (15.4%)	
Entire	15 (3.0%)	0 (0.0%)	
Histology			
WD	57 (11.4%)	11 (42.3%)	<0.001
MD	163 (32.5%)	14 (53.8%)	
PD	193 (38.4%)	1 (3.8%)	
SRC	74 (14.7%)	0 (0.0%)	
Mucinous	15 (3.0%)	0 (0.0%)	
Lauren classification			
Intestinal	211 (42.0%)	25 (96.2%)	<0.001
Diffuse	241 (48.0%)	1 (3.8%)	
Mixed	50 (10.0%)	0 (0.0%)	
Lymphatic invasion			
Absent	260 (51.8%)	15 (57.7%)	0.557
Present	242 (48.2%)	11 (42.3%)	
Vascular invasion			
Absent	445 (88.8%)	24 (92.3%)	0.756
Present	56 (11.2%)	2 (7.7%)	
Perineural invasion			
Absent	329 (65.5%)	23 (88.5%)	0.017
Present	173 (34.5%)	3 (11.5%)	
pTNM stage			
I	261 (52.0%)	17 (65.4%)	0.167
II	79 (15.7%)	4 (15.4%)	
III	139 (27.7%)	4 (15.4%)	
IV	23 (4.6%)	1 (3.8%)	
Total	502 (95.1%)	26 (4.9%)	

Abbreviations: WD, well differentiated; MD, moderately differentiated; PD, poorly differentiated; SRC, signet ring cell carcinoma; pTNM.

**Table 5:** Cox regression analysis of disease-free survival and overall survival in consecutive gastric cancer patients.

	DFS				OS			
	Univariate		Multivariate		Univariate		Multivariate	
	HR (95% CI)	*p*	HR (95% CI)	*p*	HR (95% CI)	*p*	HR (95% CI)	*p*
Age								
<65	1.00		-		1.00		1.00	
≥65	1.31 (0.92–1.86)	0.139	-	-	2.43 (1.83–3.22)	<0.001	2.90 (2.18–3.87)	<0.001
Sex								
Male	1.00		-		1.00		1.00	
Female	0.79 (0.54–1.16)	0.230	-	-	0.66 (0.48–0.91)	0.010	0.74 (0.53–1.01)	0.060
Lymphatic invasion								
Absent	1.00		1.00		1.00		1.00	
Present	9.24 (5.46–15.62)	<0.001	2.23 (1.23–4.03)	0.008	30.07 (2.27–4.14)	<0.001	1.40 (0.97–2.03)	0.076
Perineural invasion								
Absent	1.00		1.00		1.00		1.00	
Present	7.73 (5.16–11.58)	<0.001	1.45 (0.91–2.30)	0.121	3.74 (2.82–4.96)	<0.001	1.43 (0.99–2.08)	0.060
Vascular invasion								
Absent	1.00		1.00		1.00		1.00	
Present	6.49 (4.45–9.45)	<0.001	1.81 (1.23–2.66)	0.003	4.95 (3.55–6.90)	<0.001	2.11 (1.47–3.03)	<0.001
HER2 IHC								
<2+	1.00		-		1.00		-	
≥2+	0.94 (0.49–1.80)	0.852	-	-	0.90 (0.54–1.51)	0.697	-	-
*HER2* SISH								
Non-ampl	1.00		-		1.00		-	
Ampl	0.97 (0.71–1.32)	0.845	-	-	1.02 (0.61–1.70)	0.934	-	-
Trastuzumab IHC								
<2+	1.00		1.00		1.00		1.00	
≥2+	2.80 (1.31–6.01)	0.008	1.81 (0.84–3.90)	0.132	2.19 (1.08–4.44)	0.031	1.68 (0.82–3.42)	0.155
Overall *HER2* status								
Negative	1.00		-		1.00		-	
Positive	0.94 (0.42–2.15)	0.891	-	-	0.97 (0.51–1.83)	0.926	-	-
Stage								
I, II	1.00		1.00		1.00		1.00	
III, IV	19.27 (11.66–31.85)	<0.001	8.58 (4.59–16.04)	<0.001	4.96 (3.73–6.60)	<0.001	3.11 (2.06–4.68)	<0.001

Abbreviation: PFS, progression-free survival; HR, hazard ratio; CI, confidence interval; IHC, immunohistochemistry; SISH, silver in situ hybridization; Non-ampl., non-amplification; Ampl., amplification.

## Supplementary Materials

The following are available online at https://www.mdpi.com/2218-273X/9/12/782/s1, Figure S1: Results of quantum dot (QD) detection using trastuzumab and HER2 immunohistochemistry antibody before and after treatment with trastuzumab.
