# Supplementary material for: Trastuzumab Specific Epitope Evaluation as a Predictive and Prognostic Biomarker in Gastric Cancer Patients
Source: Biomolecules. 2019 Nov 26;9(12):782. doi: 10.3390/biom9120782 (PMC6995606; doi:10.3390/biom9120782)
Supplement: Supplementary file 1 [file biomolecules-09-00782-s001.pdf]

Supplementary Material

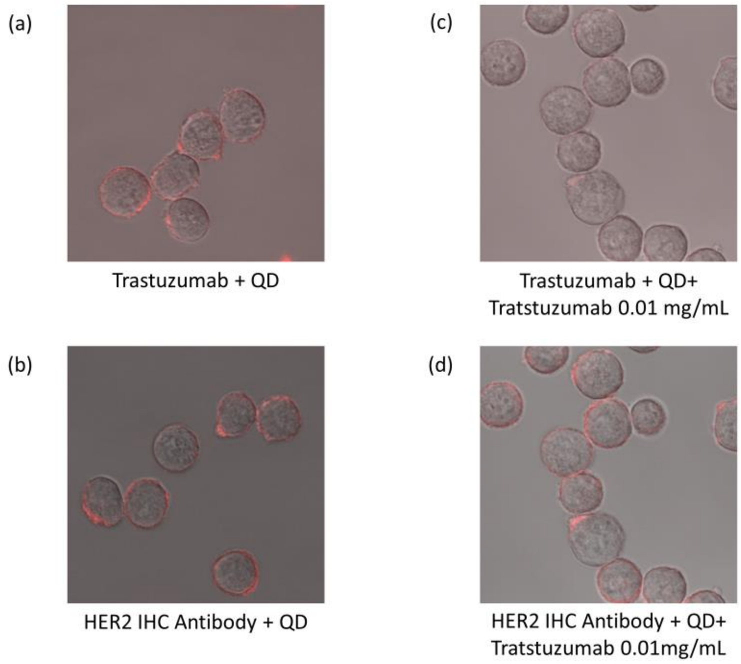

Figure S1. Results of quantum dot (QD) detection using trastuzumab and HER2 immunohistochemistry antibody before and after treatment with trastuzumab**.** Both QD labeled Trastuzumab (a) and QD labeled HER2 IHC antibody (b) detected SNU-1 cell lines in vitro. However, after treatment with 0.01 mg/mL of trastuzumab in vitro, QD labeld trastuzumab to bind to SNU-1 cellular membrane (c) in contrast to HER2 IHC antibody (d), implying that cellular imaging using trastuzumab itself better represent the actual drug binding and mechanism of action.
